# Identification of human immunodeficiency virus type 1 (HIV-1) gp120-binding sites on scavenger receptor cysteine rich 1 (SRCR1) domain of gp340

**DOI:** 10.1186/1423-0127-20-44

**Published:** 2013-07-01

**Authors:** Ying Chu, Jiahuang Li, Xilin Wu, Zichun Hua, Zhiwei Wu

**Affiliations:** 1 The Center for Public Health Research, School of Medicine, Nanjing University, Nanjing 210093, Jiangsu Province, P. R. China; 2 State Key Lab of Analytical Chemistry for Life Science, Nanjing University, Nanjing 210093, Jiangsu Province, P. R. China; 3 The State Key Laboratory of Pharmaceutical Biotechnology, School of Life Science, Nanjing University, Nanjing 210093, Jiangsu Province, P. R. China; 4 Present address: AIDS Institute, Li Ka Shing Faculty of Medicine, The University of Hong Kong, Pokfulam Hong Kong SAR, Hong Kong, People’s Republic of China; 5 Nanjing University, Meng Minwei 2104, 22 Hankou Road, Nanjing, Jiangsu 210093, P. R. China

**Keywords:** SRCR1, DMBT1, HIV-1 gp120, Automated docking

## Abstract

**Background:**

gp340, a member of scavenger receptor cysteine rich family encoded by Deleted in Malignant Brain Tumors 1 (*DMBT1*), is an important component in innate immune defense. The first scavenger receptor cysteine rich domain (SRCR1) of gp340 has been shown to inhibit HIV-1 infection through binding to the N-terminal flank of the V3 loop of HIV-1 gp120.

**Results:**

Through homology modeling and docking analysis of SRCR1 to a gp120-CD4-X5 antibody complex, we identified three loop regions containing polar or acidic residues that directly interacted with gp120. To confirm the docking prediction, a series of over-lapping peptides covering the SRCR1 sequence were synthesized and analyzed by gp120-peptide binding assay. Five peptides coincide with three loop regions showed the relative high binding index. An alanine substitution scan revealed that Asp34, Asp35, Asn96 and Glu101 in two peptides with the highest binding index are the critical residues in SRCR1 interaction with gp120.

**Conclusion:**

We pinpointed the vital gp120-binding regions in SRCR1 and narrowed down the amino acids which play critical roles in contacting with gp120.

## Background

Salivary agglutinin (SAG) and lung scavenger receptor glycoprotein (gp340) are multi-splicing products of Deleted in Malignant Brain Tumors 1 (*DMBT1*), a gene originally identified in brain tumors [1]. SAG, secreted by salivary glands, was originally found to aggregate oral *streptococcus gordonii* and *streptococcus mutans*, playing important roles in dental caries [2]. Gp340 can also bind a number of Gram-negative and Gram-positive bacteria, including *E. coli, Lactobacillus casei, Helicobacter pylori, S. aureus, S. pneumoniae, and Haemophilus. Influenzae*[3-5]. Recently, gp340 was shown to inhibit cytoinvasion of *Salmonella enterica* in intestinal epithelial cells [6] and the infectivity of influenza A virus [7]. Previous studies showed that soluble gp340 specifically inhibited HIV-1 infection [8,9], by interacting with the viral envelope gp120 [10]. Subsequent studies identified that a short sequence located at the N-terminal flank of the gp120 V3 loop interacted with gp340 [11]. An N-SRCR recombinant protein containing the first SRCR domain and one-half of the first SID of gp340 was shown to bind to the N-terminal flank of the V3 loop of gp120 and inhibit both CXCR4 and CCR5 HIV-1 isolates, exhibiting similar properties as the parental gp340 [12]. Although the bioactivities of gp340 as an innate immune component have been well documented [13], the detailed molecular mechanisms remain largely unclear. Since the SRCR1 binds to a highly conserved region of HIV-1 gp120 [12], it is a potential candidate as an anti-viral inhibitor.

SRCR consists of 90–110 amino acids and is divided into Groups A and B, depending on the number of disulfide bonds and the locations of the cysteines [13]. Group B SRCR, to which gp340 belongs, contains either 6 or 8 cysteines with cysteines at positions 1 and 4 always present while Group A SRCR contains 6 cysteines with positions 1 and 4 always absent. Since SRCR forms a highly structured domain and it is likely that structural elements are part of the gp120 binding domain, it is imperative to determine the sequence or minimal structural domain needed for mediating gp120 binding. The structure of Mac 2 binding protein (M2bp), a Group A SRCR, has been determined by crystallography [14]. In the current study, a 3-D structure of SRCR1 was constructed through homology modeling based on the known structure of M2bp, and was docked with the published 3D structure of gp120-CD4-X5 complex, which is a gp120 core in complex with soluble CD4 and a HIV-1 neutralizing antibody-X5 Fab fragment [15]. A probability analysis was conducted on the multiple docking models [16] and the gp120-contact amino acid residues on SRCR were predicted. To verify the docking analysis, linear peptides were synthesized according to the prediction values and gp120-peptide binding analysis was conducted. The results demonstrated the validity of the modeling and identified a number of regions that were in contact with gp120. This study provided atomic insight into the interactions between gp120 and SRCR1, shedding light on the inhibitory mechanism of SRCR and laid the ground for further study on the SRCR sequence/structures important for HIV-1 inhibition and the design of new HIV-1 target drugs.

## Methods

### Modeling of SRCR1 domain

The structure of SRCR1 (residues 95–203 on *DMBT1*, renumbered as 1–109 in this article) was built based on the known structure of M2bp SRCR domain (PDB ID: 1BY2) using software MODELLER9v4 [14,17-19]. Structural refinements were accomplished by energy minimization and molecular dynamics using software Insight II 2001 (Accelrys, San Diego, CA).

### Automated docking of SRCR1 to monomeric gp120-CD4-X5 complex

Docking of SRCR1 to the gp120-CD4-X5 complex (PDB ID: 2B4C) was performed using ZDOCK3.0.1, a rigid-body protein-protein docking software [20]. ZDOCK used a fast Fourier transformation to search all possible binding modes for the proteins, performing evaluation based on shape complementarity, desolvation energy, and electrostatics. The top predictions from ZDOCK were then recomputed by RDOCK to improve the energies and eliminate clashes.

### Peptide design, synthesis and purification

A complete set of 15-mer peptides, overlapping by 10 amino acids, was derived from SRCR1. These peptides were numbered according to the SRCR1 sequence, P1 standing for 1–15 residues, P2 for 5–20 and so on. To study the role of the vital amino acids for gp120 binding, a set of peptides was introduced alanine substitutions in corresponding residues. All peptides were synthesized and purified by high-performance liquid chromatography (HPLC) to >95% purity (HD Biosciences Co., Shanghai, China). The biotin tag was labeled at the N-terminus of the peptide.

### Peptide binding assay (solid-phase ELISA)

Recombinant gp120s were purchased from Immuno Diagnostics, Inc (Woburn, MA, USA) or from the NIH AIDS Reagent Program (Bethesda, MD, USA). 96-well polyvinylchloride plates (Corning, NY, USA) were coated with 50 μl of 8ug/ml gp120 diluted in 50 mM bicarbonate buffer, pH 9.6, and incubated at 4°C for 16 hours. Unbound protein was removed by repeated washing with 20 mM Tris–HCl, pH7.4, containing 0.05% Tween-20 (washing buffer). Nonspecific sites were blocked with 200 μl 2% nonfat milk dissolved in washing buffer, at 37°C for 60 min. 50 μl of 30 μg/ml biotinylated peptides diluted in washing buffer were added and allowed to bind gp120 at 37°C for 60 min. Unbound peptides were removed by repeated washing. For competition studies, biotinylated P19 (40 μg/ml) was mixed with increasing concentrations of non-biotinylated P19, added to a gp120-coated plate (8ug/ml) and incubated at 37°C for 60 min. Unbound peptides were removed by repeated washing. Since P1 showed no binding to gp120 in the previous binding analyses, it was used as a control at the maximum concentration (640 μg/ml). The bound peptides were detected with AP-conjugated streptavidin (Vector, CA, USA) and PNPP liquid substrate (Sigma, USA), and measured at 405 nm.

## Results and discussion

### Structural modeling of SRCR1

**Figure 1:**
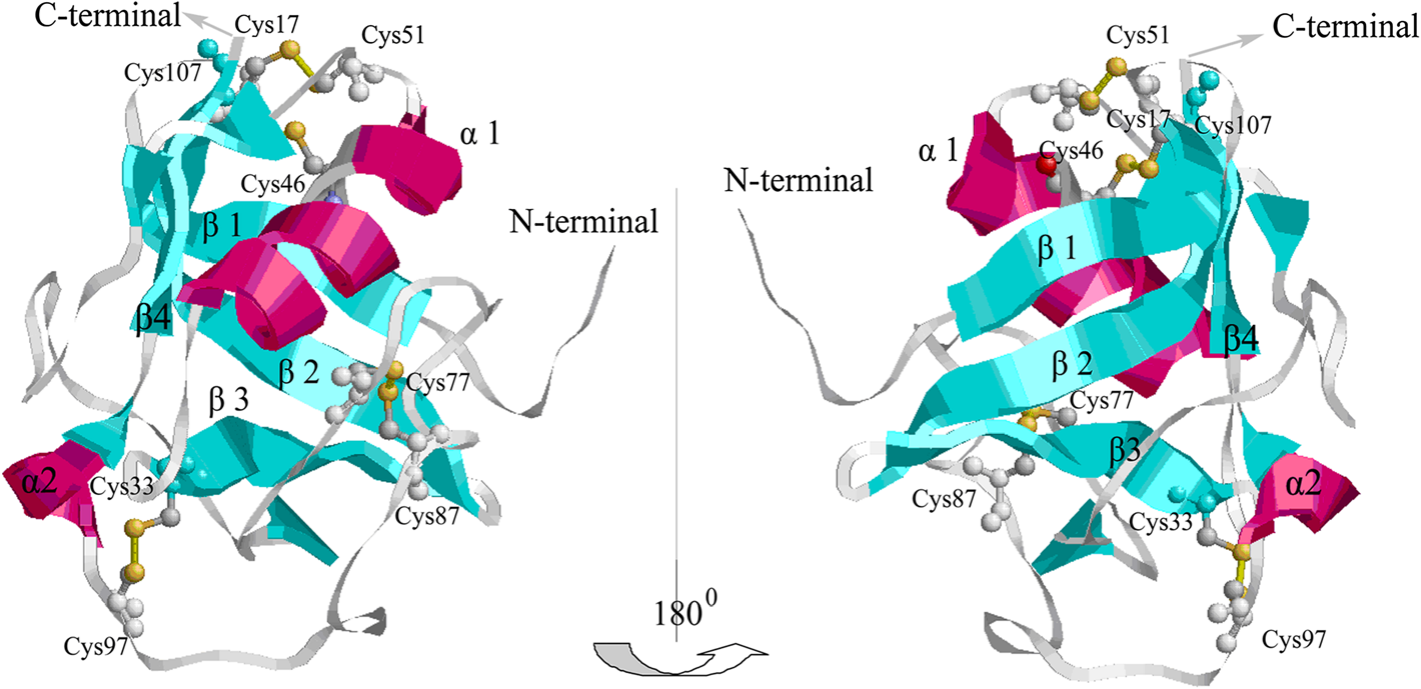
**Two reverse views of SRCR1 structure.** β-strands are in cyan; two α-helices are in magenta. Disulfide bridges are in yellow and the sequence numbers of cysteine residues are indicated.

*DMBT1* SRCR1 and M2bp SRCR shared high homology (50%) in primary sequence. The SRCR1 3D structure was organized around a curved four-stranded β-sheet cradling two α-helices (Figure 1). There were 4 disulfide bridges in SRCR1: Cys33 and Cys97 forming a disulfide bridge that linked the C-terminus of the β3 strand to the α2-helix; the Cys46-Cys107 disulfide bridge linking the α1-helix to the underlying β-sheet; the Cys77-Cys87 disulfide bridge circularizing the loop and containing a turn around Glu81; and the Cys17-Cys51 disulfide bridge linking the α1-helix to the β-turn between the β1 and β2 strands.

Comparing to M2bp SRCR, the SRCR1 has an additional disulfide bridge Cys17–Cys51 while the corresponding M2bp residues, Asn15 and Phe49, are in close proximity with a Cα–Cα distance of 7.5 Å [14]. Hohenester et al. postulated that the 1–4 disulfide bridge (Cys17–Cys51) in group B domains (SRCR1) would link the C-terminus of the α-helix (α1) to the A–B loop (β1-β2) and packed favorably against the 3–8 bridge (Cys46–Cys107) [14]. The disulfide bond Cys17–Cys51 was therefore inserted in the structure according to their hypothesis (Figure 1). The surface loop structure with a β-turn, located between β1 and β2 strands, was previously shown to be involved in bacteria binding [4].

### Docking of SRCR1 with monomeric gp120-CD4-X5 complex

**Figure 2:**
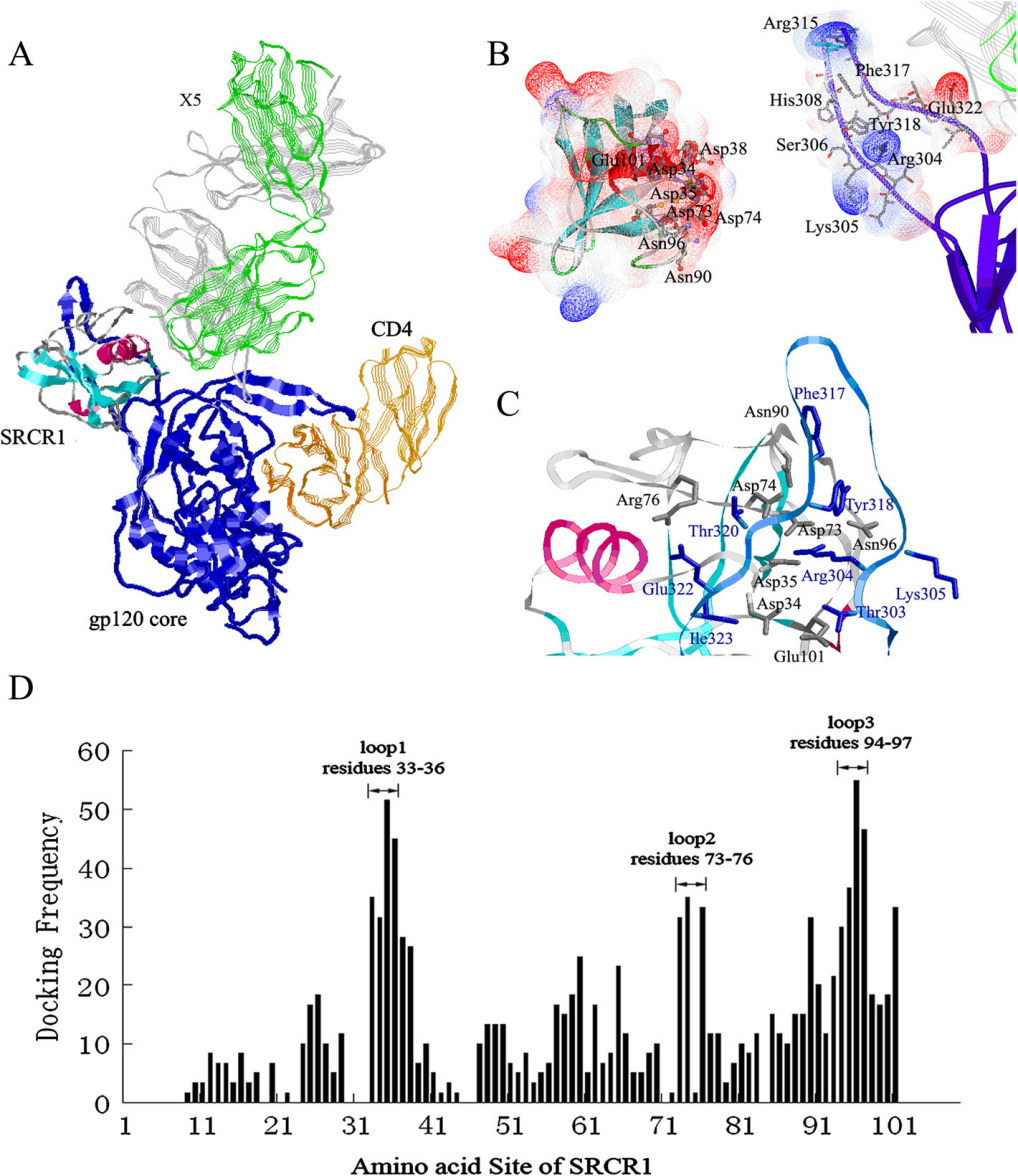
**Docking of SRCR1 with monomeric gp120-CD4-X5 complex. A)** The structure of the docked SRCR1. SRCR1 and the gp120 core (in blue) are shown in solid ribbon style. CD4 and X5 are represented in silk ribbon style with orange (CD4), green (X5 light chain) and grey (X5 heavy chain), respectively; **B)** Electrostatic surface of SRCR1 (left) and the V3-loop of gp120 (right). The molecular surfaces of both SRCR1 and the V3-loop of gp120 are colored according to the calculated electrostatic surface potential with negative charges in blue, neutral in white, and positive charges in red; **C)** The closer view of the gp120-binding sites. The residues in SRCR1, which make contact with gp120, are shown as sticks in grey, whereas the residues in gp120 are colored in blue; **D)** The docking frequency of residues 1–109 in SRCR1. Three high docking frequency loop regions 33–36, 73–76 and 94–97 are indicated.

Our early study demonstrated that gp340 binding to gp120 was significantly enhanced by the pre-treatment of gp120 with soluble CD4 and that the 17b antibody binding to the gp120-sCD4 complex did not abrogate gp340 binding and vice versa [11]. We suggested that the gp340-binding region on gp120 was occluded or partially occluded on the native gp120 and sCD4 binding induced the exposure of the region, thus allowing high affinity interaction between gp340 and gp120 [11]. Therefore, we speculated that a gp120 in complex with sCD4 would be a favorable model for our docking analysis, Since the gp120-sCD4-17b structure does not contain a V3 sequence [21], a newly published crystal structure of a V3-containing HIV-1 gp120 core in complex with sCD4 and a HIV-1 neutralizing antibody-X5 [15] was selected. X5 and 17b belong to the same class of HIV-1 neutralizing antibodies termed CD4-induced (CD4i) antibodies, which recognize highly conserved epitopes exposed upon CD4 binding. The binding of the CD4i antibodies to gp120 is typically enhanced by the CD4 binding. To provide restrains on docking possibilities, a number of factors were considered. First, the location of the V3 loop and the surface for SRCR1 binding on the trimeric viral spike in native state, as determined by Liu et al. [22], were used to orient the SRCR1 structure in the docking. Second, gp340 is a macromolecule with 14 SRCR domains in tandem order, thus SRCR1 can only be docked to a region outside of the plane formed by the β-sheet on the V3 loop due to the steric hindrance. Figure 2A showed a typically docked complex of SRCR1 and gp120-sCD4-X5. The SRCR structure interacted with gp120 on the open face, near the V3 loop site [11], and the N- and C-termini of SRCR, which link the SRCR1 subunit to the rest of the gp340, were pointed away from the gp120.

### Analysis of gp120-contacting amino acids on SRCR1

The docked complex of SRCR1 and gp120-CD4-X5 was shown in Figure 2A. Three regions with apparently high docking frequencies were identified (Figure 2D): the surface Loop1 between the β3 strand and α1-helix (Cys33 to Ser36), the surface Loop2 near the Cys77-Cys87 disulfide bridge (Asp73 to Arg76) and the surface Loop3 near the α2-helix (Ser94 to Cys97). These three loops formed a negatively charged cluster on the SRCR1 3D structure and a number of amino acids contacted with the V3-loop of gp120 as shown in Figure 2B. Figure 2C showed a closer view of gp120-binding sites on SRCR1. Frequently, Asp35 and Asp73 formed salt bridges with Arg304 of gp120. Ser36 formed a Van der Waals interaction or a hydrogen bond with Thr320. Asp74 formed a hydrogen bond with Tyr318 and Thr320. Arg76, the only basic residue in the gp120-binding sites, frequently formed a salt bridge with Glu322 of the V3 loop. Asn96 showed the highest binding frequency among contact residues in SRCR1(Figure 2D), and always made contact with Lys305, Arg304, Ser306 and Tyr318. Ser94 and His95 frequently interacted with Tyr318. Docking analysis indicated that the frequency of gp120 interaction with the residues 93–97 in SRCR1 was higher than that of any other residues, suggesting that this region might be the most important for the stability of the interaction.

xThe amino acid contacting frequency analysis was consistent with published studies [11]. Earlier studies indicated that the contribution of the charges of V3 residues dominated gp120 electrostatic potential [23] and a minimal gp340-binding sequence was localized to the V3 N-terminal flank (aa303-306), which was rich in positively charged residues [11]. A V3 peptide containing the residues 303–306 was found to bind both soluble [11] and cell-associated gp340 [24], and inhibited gp120-gp340 interaction. In addition, our model also suggested that Phe317, Tyr318, Thr320 and Glu322 at the C-terminus of the V3 were involved in gp120-SRCR interaction, though ELISA failed to show such an interaction [11]. Cys33, Asp34, Asp35, Asp73, Asp74, Arg76, His95, Asn96, and Cys97 were conserved in all 13 SRCRs of gp340, indicating that they might be indispensable for the bioactivity of gp340 and that the other SRCRs might also bind gp120. In some SRCR domains, Ser36 and Ser94 were replaced by Thr and Tyr, respectively, which did not significantly change the polarity of the residues. Therefore, we speculated that inter-molecular salt bridges and polar interactions were conserved and important for the specificity of the interaction.

### Gp120 interaction with SRCR1 peptides

**Figure 3:**
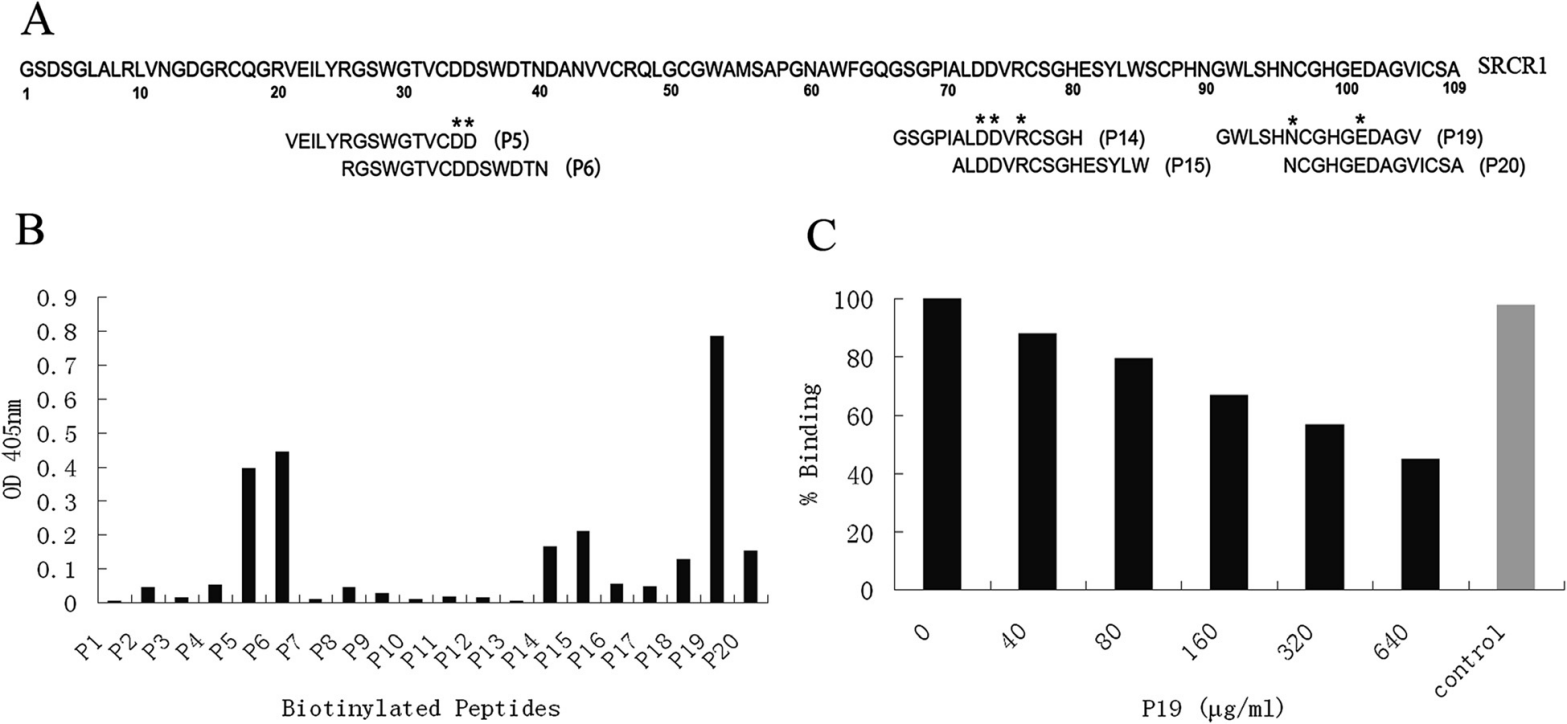
**Gp120 interaction with SRCR1 peptides. A)** The schematic illustration of SRCR1 sequence. Twenty overlapping 15-mer peptides covering the complete SRCR1 sequence were designed and synthesized. These peptides were numbered according to SRCR1 sequence, P1 standing for 1–15 residues, P2 for 5–20 and so on. The peptides with highest binding index with gp120 were identified and the key binding amino acids were marked with star; **B)** Recombinant gp120_BaL_ interaction with peptides derived from SRCR1. Biotinylated peptides (30 μg/ml) was allowed to bind immobilized gp120 (8 μg/ml) and were tested for their binding capability to gp120 with ELISA; **C)** Nonbiotinylated P19 competitively inhibited biotinylated P19 binding to gp120 (8 μg/ml). Biotinylated P19 (40 μg/ml) was pre-incubated with increasing concentrations of non-biotinylated P19, added to a gp120-coated plate (8ug/ml) and incubated at 37°C for 60 min. Unbound peptides were removed by repeated washing. Since P1 showed no binding to gp120 in previous binding assays, it was used as a control at the maximum concentration (640 μg/ml).

To validate the predictions of the docking analysis and to characterize the gp120-binding region, a series of linear peptides spanning SRCR1 sequence were synthesized and analyzed for their interactions with gp120 in a solid-phase ELISA. The sequence of SRCR1 was showed in Figure 3A and the peptides with high binding index were identified according to their location in the sequence of SRCR1. Figure 3B showed that there were a number of over-lapping peptides interacting with gp120 and with P5, P6 and P19 showing the highest binding indexes. The sequence shared by P5 and P6 was RGSWGTVCDD, which included the high docking frequency residues Cys33, Asp34 and Asp35 within the Loop 1. P19, containing the high docking frequency residues Asn96 and Glu101 in Loop 3, exhibited the highest binding index. P14 and P15, with an overlapping sequence ALDDVRCSGH and high docking frequency residues Asp73, Asp74 and Arg76, also interacted with gp120. The results were consistent with the computer modeling and summarized in Table 1.

**Table 1:** SRCR1 peptides binding to gp120

**Peptide**	**Amino acid sequence**	**Residue of SRCR1 sequence**	**Relative binding**
P5	VEILYRGSWGTVCDD	21-35	+4
P6	RGSWGTVCDDSWDTN	26-40	+4.5
P14	GSGPIALDDVRCSGH	66-80	+1.5
P15	ALDDVRCSGHESYLW	71-85	+2
P19	GWLSHNCGHGEDAGV	91-105	+7.5
P20	NCGHGEDAGVICSA-	96-109	+1.5

To demonstrate the specificity of the interaction, biotinylated P19 binding to gp120 was competed with a nonbiotinylated P19 in an ELISA. Figure 3C showed that increasing concentrations of nonbiotinylated P19 reduced the biotinylated P19 binding to gp120 in a dose-dependent manner while the control P1 had no effect, suggesting that the P19-gp120 interaction was sequence specific and not mediated by the biotin.

**Figure 4:**
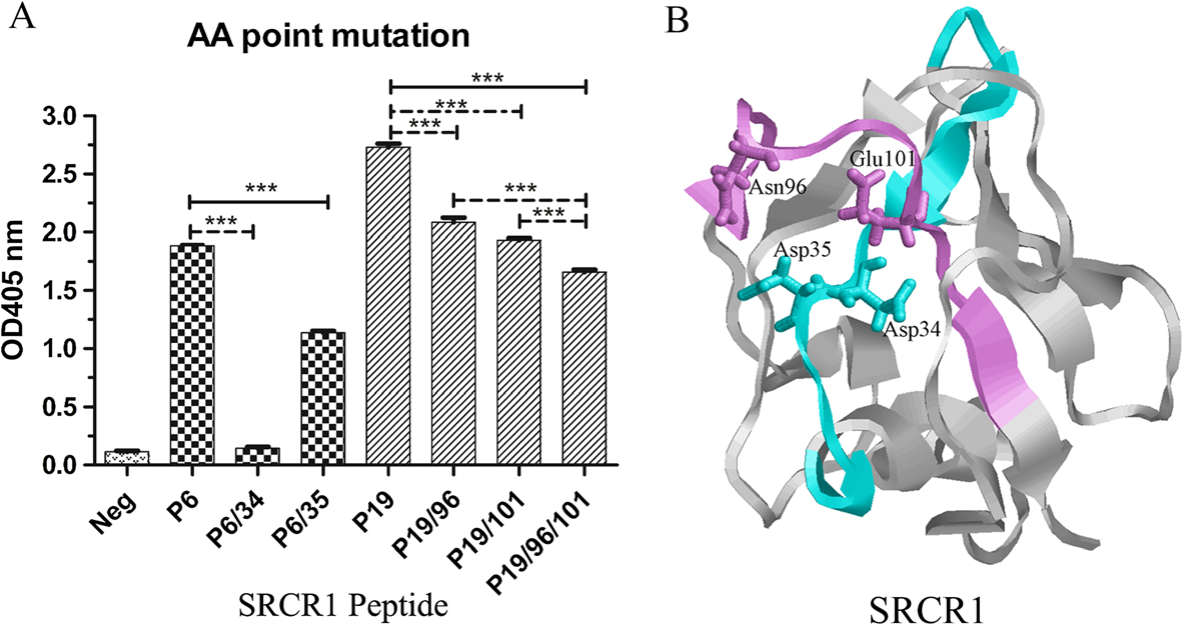
**Determination of critical residues in P6 and P19 peptides. A)** Alanine substitution scan of the peptides. Biotinylated peptides (30 μg/ml) were allowed to bind immobilized gp120_BaL_ (8 μg/ml). P6/34 means that Asp34 in P6 was substituted by alanine. It was same to other peptides, such as P6/35, P19/96, P19/101 and P19/96/101; P1 was used as a negative control; **B)** The structural view of P6 and P19 in SRCR1 model. The P6 was colored in cyan and P19 was colored in purple. Asp34, Asp35, Asn96 and Glu101 residues were labeled on the regions of P6 and P19.

To further confirm the docking frequency, we introduced alanine substitutions into four residues with the highest docking frequency in two peptides (Asp34 and Asp35 in P6, Gln96 and Glu101 in P19), either individually or in combination. All the mutant peptides showed reduced binding with gp120 (Figure 4A). However, the P19 binding to gp120 was minimally affected by mutations at 96 and 101, respectively, or in combination. We think that this may reflect the discrepancy between the modeling and the peptide-based binding assay. The modeling was performed on a folded SRCR1 with three surface loop regions directly interacting with gp120. Asn96 and Glu101, both located in the interface of the SRCR1-gp120 docking complex (seeing Figures 2B and 4B), were thus predicted to be important for protein interaction. However, these two amino acids in P19 may not be in the best conformation to interact with gp120 and some other amino acids that are not located in the interface may be available in P19 to interact with gp120.

By using a serial of peptides based on a consensus SRCR sequence, a bioactive peptide (SRCRP2) was shown to bind streptococcal and mediated bacterial aggregation [4]. Interestingly, the sequence of SRCRP2 coincides with the sequences of P5 and P6. Thus we hypothesized that the same region of SRCR1 contributed to the binding of both the bacterial and HIV-1. However, P5 and P6 didn’t show significant inhibitory effect in an in vitro HIV-1 pseudovirus inhibitory assay (data not shown). We also discovered that disulfide bond-disrupted SRCR1 protein had significantly reduced binding to gp120 (data not shown). In view of the tightly folded SRCR domain, we speculated that a certain conformational element is required for SRCR1 to form high affinity interaction with gp120 and to inhibit HIV-1 infection, consistent with the current observations that the gp120-contacting amino acids were distributed in the distal sequence. Based upon our modeling, P6 and P19 were linked with a disulfide bridge formed by Cys33 and Cys97. Thus, distal residues (Asp34, Asp35, Asn96 and Glu101) along the linear sequence were brought to proximity by disulfide bonds and formed a gp120 binding plain (Figure 4B). The structural element required for SRCR1 to form high affinity binding with gp120 and for inhibiting viral infection needs further investigation. The identification of the contacting amino acid residues in the current study will facilitate our understanding of the structure and the design of molecular mimicry expressing the needed conformation as a more effective drug.

## Conclusions

We performed docking analysis on gp120-SRCR1 interaction and made a number of predictions on the SRCR1 amino acid residues that may interact with gp120. Binding analysis using a series of synthetic peptides derived from SRCR1 demonstrated that three high binding loop regions of aa33-36, 73–76 and 94–97 on SRCR1 bound gp120. The relative binding indexes were consistent with the predictions of the docking model, suggesting that our model is appropriate for further analysis of SRCR1-gp120 interaction and that, by combining biochemical approaches, the model may facilitate the designing of new HIV-1 target drugs.

## Competing interests

The authors declare that they have no competing interests.

## Authors’ contributions

YC and JHL carried out overall research work; YC and XLW carried out the peptide binding assay; YC, JHL, ZCH and ZWW conceived and designed the experiments; YC and ZWW drafted the manuscript. All authors have read and approved the final manuscript.
